# Poor Compliance with Sepsis Guidelines in a Tertiary Care Children’s Hospital Emergency Room

**DOI:** 10.3389/fped.2018.00053

**Published:** 2018-04-05

**Authors:** Benjamin Louis Moresco, Clinton Woosley, Morris Sauter, Utpal Bhalala

**Affiliations:** ^1^Baylor College of Medicine, Houston, TX, United States; ^2^The Children’s Hospital of San Antonio, San Antonio, TX, United States

**Keywords:** severe sepsis, septic shock, management, adherence, guidelines

## Abstract

**Objectives:**

This study aimed to assess factors related to adherence to the Pediatric Advanced Life Support guidelines for severe sepsis and septic shock in an emergency room (ER) of a tertiary care children’s hospital.

**Methods:**

This was a retrospective, observational study of children (0–18 years old) in The Children’s Hospital of San Antonio ER over 1 year with the International Consensus Definition Codes, version-9 (ICD-9) diagnostic codes for “severe sepsis” and “shocks.” Patients in the adherent group were those who met all three elements of adherence: (1) rapid vascular access with at most one IV attempt before seeking alternate access (unless already in place), (2) fluids administered within 15 min from sepsis recognition, and (3) antibiotic administration started within 1 h of sepsis recognition. Comparisons between groups with and without sepsis guideline adherence were performed using Student’s *t*-test (the measurements expressed as median values). The proportions were compared using chi-square test. *p*-Value ≤0.05 was considered significant.

**Results:**

A total of 43 patients who visited the ER from July 2014 to July 2015 had clinically proven severe sepsis or SS ICD-9 codes. The median age was 5 years. The median triage time, times from triage to vascular access, fluid administration and antibiotic administration were 26, 48.5, 76, and 135 min, respectively. Adherence to vascular access, fluid, and antibiotic administration guidelines was 21, 26, and 34%, respectively. Appropriate fluid bolus (20 ml/kg over 15–20 min) was only seen in 6% of patients in the non-adherent group versus 38% in the adherent group (*p* = 0.01). All of the patients in the non-adherent group used an infusion pump for fluid resuscitation. Hypotension and ≥3 organ dysfunction were more commonly observed in patients in adherent group as compared to patients in non-adherent group (38 vs. 14% *p* = 0.24; 63 vs. 23% *p* = 0.03).

**Conclusion:**

Overall adherence to sepsis guidelines was low. The factors associated with non-adherence to sepsis guidelines were >1 attempt at vascular access, delay in antibiotic ordering, fluid administration using infusion pump, absence of hypotension, and absence of three or more organs in dysfunction at ER presentation.

## Introduction

**Figure 1:**
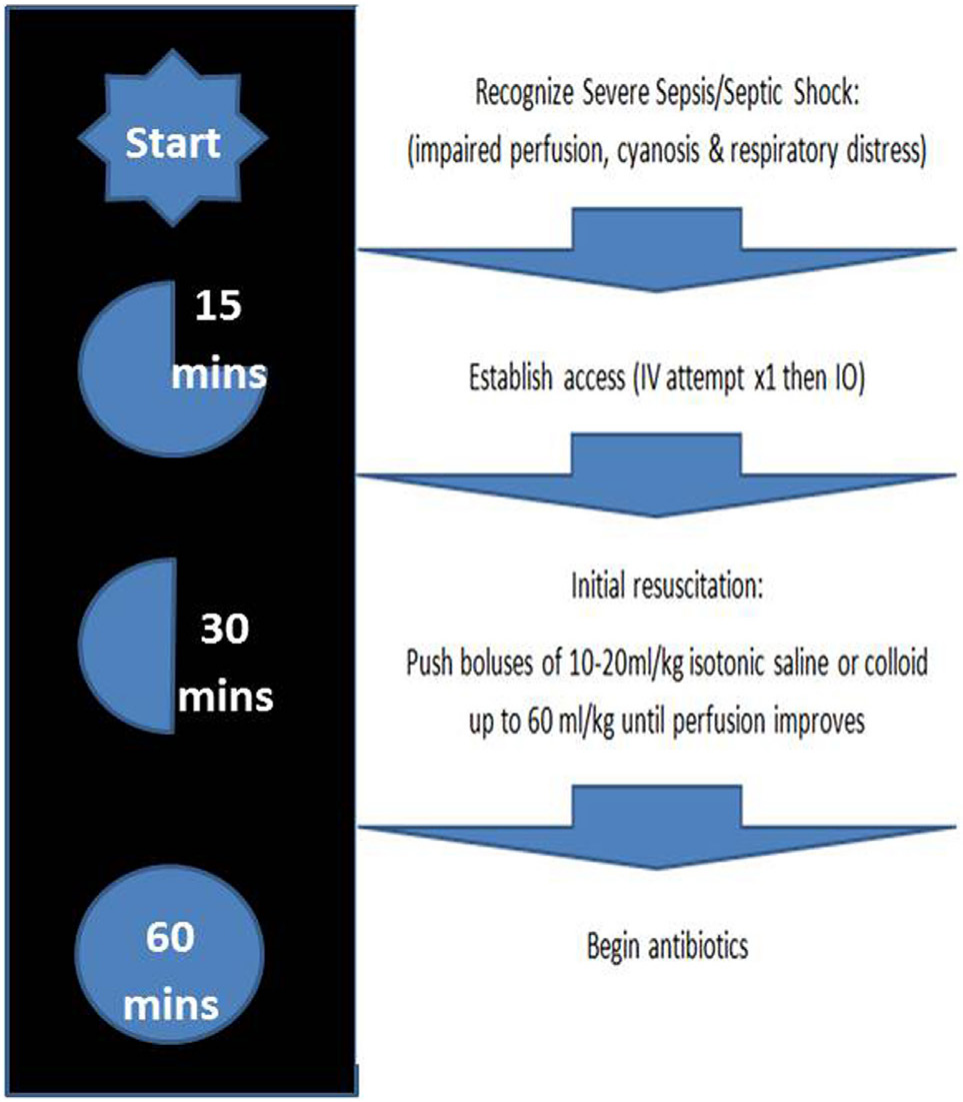
Management of severe sepsis/septic shock based on 2015 Pediatric Advanced Life Support guidelines (4).

Severe sepsis and septic shock (SS) in children represent significant challenges for all pediatricians and emergency care providers, and immediate and aggressive treatment is needed. With a high overall mortality rate of 8.9–25%, and likely higher rate in those with chronic illness, they represent a leading cause of death among infants and children (1–4). However, it should be noted that physician guided diagnosis of SS has a concordance by research and clinical definitions of only 42.6% (5). The Pediatric Advanced Life Support (PALS) and Surviving Sepsis guidelines recommend early recognition of SS, early administration of antibiotics (grade 1D), and early administration of isotonic crystalloid or colloid fluid (grade 2C). According to the Guidelines, vascular access should be obtained within 5–10 min of recognition of SS, followed by rapid administration of antibiotics and fluid resuscitation with boluses of up to 20 ml/kg over the course of 5–10 min titrated to reversing hypotension, increasing urine output, and attaining normal capillary refill, peripheral pulses, and level of consciousness without inducing hepatomegaly or rales (usually up to three fluid boluses before considering inotropes), all within an hour of recognition of SS (6, 7) (Figure 1).

In a study done at Boston Children’s Hospital using the electronic medical record (EMR) data, overall adherence to PALS SS guidelines was found to be only 19% (3). They used a sepsis bundle consisting of five components, namely (a) early recognition of SS, (b) obtaining vascular access, (c) administering intravenous fluids, (d) delivery of vasopressors for fluid refractory shock, and (e) antibiotic administration, based on earlier studies, to evaluate adherence. However, when patients were managed within the guideline’s recommendations, they demonstrated that the number of cases of SS between each death from this condition increased, demonstrating significant outcome improvement (8). Sufficient data on factors or processes associated with non-adherence to the sepsis guidelines are lacking. In our institution, the sepsis Quality Improvement (QI) team reviewed a random selection of patients who presented to the ER from July 2014 to July 2015 with the ICD-9 code of SS. The initial QI review from the EMR suggested a significant delay in components of sepsis intervention in our pediatric ER, which prompted us to study the factors related to the poor adherence to the sepsis guidelines in our ER. We sought to determine the extent of non-adherence to pediatric sepsis guidelines in our ER with regard to (a) early recognition of sepsis, (b) time to administration of first antibiotic, (c) time to initiation of first fluid bolus, (d) rate of fluid bolus, and (e) other factors associated, in order to identify and remedy factors contributing to non-adherence, as well as provide insights for other institutions.

## Materials and Methods

### Study Design and Selection of Participants

The study was conducted in the ER at The Children’s Hospital of San Antonio (CHofSA), a freestanding, 200-bed, tertiary care center with more than 80,000 ER visits annually. The study was approved by Baylor College of Medicine institutional review board and CHofSA feasibility committee. Due to retrospective nature of the study, our IRB approved the study with a waiver of informed consent.

The study was a retrospective chart review of children (0–18 years old) with the ICD-9 diagnostic codes for “severe sepsis” and “SS” admitted to our children’s hospital ER over the course of 1 year (July 2014 to July 2015). Since our children’s hospital ER does not maintain a case log for SS patients, the best way to identify these cases retrospectively was using ICD-9 codes. Patients were included in the study if they met the international pediatric sepsis consensus conference definition of SS by manual chart review (9, 10). Patients were excluded if (a) they either presented or progressed to cardiopulmonary arrest in triage or ER due to limited data relative to their sepsis management or (b) if they did not meet criteria for systemic inflammatory response syndrome (SIRS) at triage or (c) developed SS after their ER stay. Those patients who progressed to cardiopulmonary arrest in triage or the ER had a change in priority from sepsis management to CPR. Therefore, the only document available for these patients was the code sheet and we excluded them from the study.

### Definition of Terms

Patients in the adherent group were those who met all three elements of PALS guideline adherence: (1) rapid vascular access with at most one IV attempt before seeking alternate access (unless already in place), (2) fluids administered within 15 min from sepsis recognition, and (3) antibiotic administration started within 1 h of sepsis recognition.

Organ dysfunction and SS definitions were established using the 2005 international pediatric sepsis consensus conference (9). *Cardiovascular system dysfunction* was defined as low blood pressure for age or need for vasoactive drugs to maintain blood pressure or two of the following: unexplained metabolic acidosis (base deficit > 5.0 mEq/L) and/or increased arterial lactate (>2 times the upper limit of normal) and/or oliguria (<0.5 mL/kg/h) and/or prolonged capillary refill (>5). *Respiratory system dysfunction* was defined as PaO_2_/FiO_2_ < 300, or PaCO_2_ > 65, or need for >50% FiO_2_, or need for non-elective invasive or noninvasive mechanical ventilation. *Neurologic system dysfunction* was defined as a Glasgow Coma Score (GCS) ≤11 or acute change in mental status with decrease in GCS ≥3 points from an abnormal baseline. *Hematologic system dysfunction* was defined as a platelet count of <80,000/mm^3^ or an INR > 2 or additional *a priori* definitions of any elevated or suppressed white blood cell count [>20 × 10(9)/L or <4 × 10(9)/L] or any suppressed red blood cell count (hemoglobin < 7 g/dL). *Renal system dysfunction* was defined as a serum creatinine ≥2 times the upper limit of normal for age or a twofold increase in baseline creatinine or hyperkalemia (potassium level > 7 mmol/L). *Hepatic system dysfunction* was defined as a total bilirubin of ≥4 mg/dL or alanine transaminase level of two times the upper limit of normal for age. In addition, we described gastrointestinal, endocrinologic and endothelial dysfunction *a priori*. Since gastrointestinal, endocrinologic and endothelial dysfunction has not been defined in the literature, we defined gastrointestinal, endocrinologic and endothelial dysfunction *a priori* based on clinical and laboratory derangement in the respective system. Gastrointestinal system dysfunction, defined as a rigid abdomen or severe vomiting/diarrhea on presentation; endocrinologic system dysfunction, defined as an elevated lactate dehydrogenase (>380 U/L) or any metabolic acidosis (anion gap > 12); and endothelial system dysfunction, defined as a low level of fibrinogen (<150 mg/dL).

Although the nomenclatures for sepsis and SS are changing, these changes have not yet impacted pediatrics. For this reason, the sepsis definitions are based on the 2005 international pediatric sepsis consensus conference are as follows (9).

#### Sepsis

Of the 4 SIRS criteria, ≥2 (temperature, heart rate, respiratory rate, leukocyte count), one of which must be abnormal temperature or leukocyte count in the presence of a suspected or proven infection.

#### Severe Sepsis

Sepsis + cardiovascular dysfunction OR acute respiratory distress syndrome OR two or more other organ dysfunctions as defined above.

#### Septic Shock

Sepsis and cardiovascular organ dysfunction as defined above.

### Evaluation of Adherence to Guidelines

Our EMR was indexed to evaluate adherence to the 2015 PALS Guidelines which are the guidelines followed by our institution for the management of SS (6). For all the eligible children, EMR data were collected on demographics, triage vitals, organ system involvement, time to vascular access, time to administration of first antibiotic, time to initiation of first fluid bolus, and rate of fluid administration. In our institution, as soon as the patient is first seen in the ER triage, a set of vitals are obtained and a triage score is assigned to indicate acuity of the patient. Then, the patient is moved to the main ER area where the ER attending sees the patient. In our study, the triage time was defined as the time spent in the ER triage before being seen by an ER attending. Front-line provider (FLP) time indicates when the ER practitioner placed the first order for a patient. Time to vascular access was defined as the time when nurse recorded in the nursing documentation that an IV was placed. *Fluid- and antibiotic-order times* were defined as the respective time when orders were placed by the FLP and *fluid- and antibiotic-administration times* were defined as the respective times when fluid and antibiotic were started. Times were gathered from triage notes, nursing documentation, pharmacy documentation, and medication administration times as recorded on the EMR and cross-referenced with order entries to confirm validity. The timing of when antibiotic doses were removed from the automated medication dispensing system (AMDS) was determined by the pediatric intensive care unit (PICU) pharmacist. The patient’s account number was used to review the admission in our EMR, and the exact time of the AMDS vend was recorded for all antibiotics removed from the ER AMDS machine. The time from triage to FLP was considered surrogate for time to sepsis recognition. Patients were subdivided into the adherent group (antibiotics administered within 1 h from FLP, ≤1 attempt at vascular access and fluid administered within 15 min from FLP) and the non-adherent group (meeting neither fluid nor antibiotic PALS-sepsis guideline recommendations).

### Factors Related to Non-Adherence

Delay in recognition, use of infusion pump for fluid bolus, and laboratory tests used to classify organ dysfunction in SS were determined using the EMR. In addition, fluid order, and administration time; rate of infusion pump; antibiotic order and administration times; patient demographics; and patient disposition were also determined based on review of the EMR. In order to understand the factors related to non-adherence in antibiotic administration we reviewed the data from the AMDS. Apart from reviewing the AMDS data we also determined that all the antibiotics are available from the AMDS and ER nurses are expected to remove them and prepare them for administration. In order to review delay in vascular access we evaluated the number of IV attempts as documented in nursing notes. As a surrogate for FLP we used first order-entry time for the physician in the ER.

### Statistical Analysis

Statistical analysis was performed using Stata version 12 (StataCorp LLC, College Station, TX, USA). Comparisons between groups with and without sepsis guideline adherence were performed using Student’s *t*-test (the measurements expressed as median values). The proportions were compared using chi-square test. *p*-Value ≤0.05 was considered significant.

## Results

Fifty-eight patients were identified as meeting ICD-9 coded “severe sepsis” or “SS” from July 2014 to June 2015. These records were screened for any exclusion criteria. Ten patients from the original cohort were removed because they did not meet SIRS criteria at triage, and five were removed because they either presented in cardiorespiratory arrest or arrested in the ER prior to transfer to inpatient or the PICU. Thus, 43/58 (74%) patients were included in the final analysis. During the study time-period our children’s hospital did not have a residency program and therefore, there were no residents in the ER and all the patients were triaged to a triage level where nurse practitioners were not the first line providers. All the patients in our study were managed by ER physicians.

**Figure 2:**
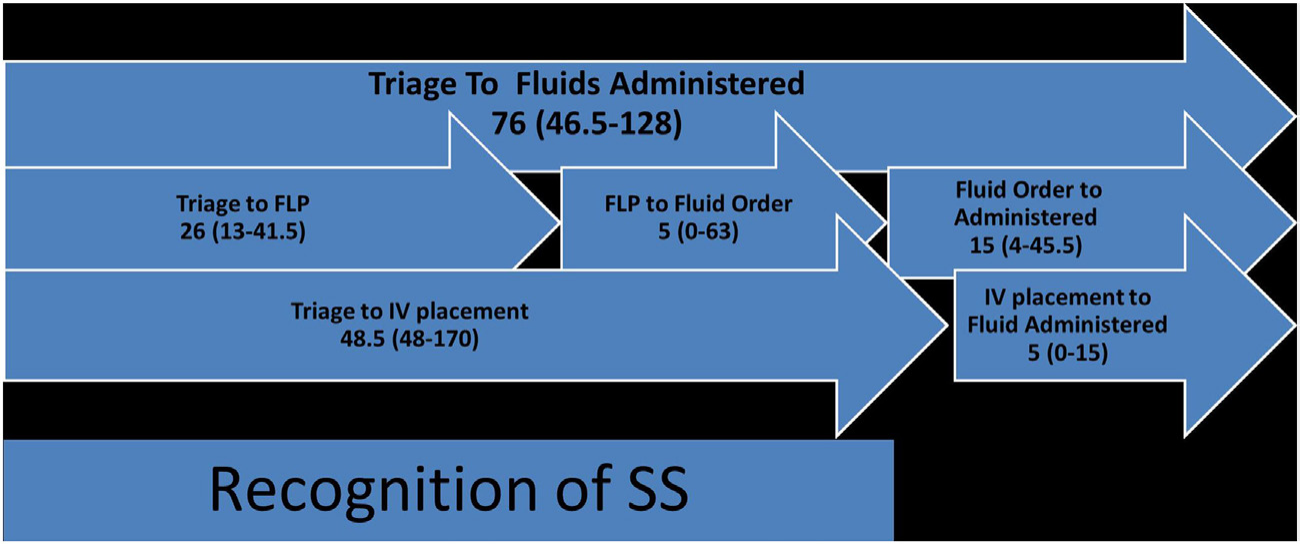
Fluid and IV time course (FLP, front-line physician, usually ER-physician) (each numerical value in the figure represents time in minutes).

**Figure 3:**
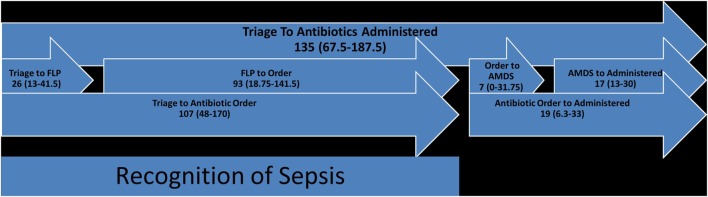
Antibiotic time course (FLP, front-line physician, usually ER-physician; AMDS, automated medication dispensing system) (each numerical value in the figure represents time in minutes).

The median age was 5 years (IQR 25–75%: 0.4–12.5 years). Of the 43 (88%) patients, 38 met the definition for SS on presentation, whereas 5/43 (12%) patients progressed to SS during their stay in the ER. Of the 43 (9%) patients, 4 died during the time period evaluated. The proportion of patients with individual organ dysfunction has been summarized in Table 1. The median triage time (time from triage to FLP) and times from triage to vascular access, fluid administration, and antibiotic administration were 26, 48.5, 76, and 135 min, respectively (Figures 2 and 3). The guideline-adherence rates to rapid vascular access and timely administration of antibiotics and appropriate fluid bolus were 21, 34, and 26%, respectively. Eight patients (19%) were in the adherent group (antibiotics administered within 1 h and fluids administered within 15 min from sepsis recognition or FLP time), and 35/43 (81%) patients were in the non-adherent group.

**Table 1:** Patient demographics.

Patient characteristic	*n*(%); (*n* = 43)
Male	23 (53)
Age [median (IQR 25–75%)] (years)	5 (0.4–12.5)
Mortality	4 (9)
Organ dysfunction	
Neurologic	7 (16)
Respiratory	19 (44)
Cardiovascular	27 (63)
Gastrointestinal/Hepatic	13 (30)
Endocrine	17 (40)
Endothelial	4 (9)
Renal	13 (30)
Hematologic	9 (21)
Guideline adherent group	8 (19)
Guideline non-adherent group	35 (81)

**Independent factor related to guideline adherence**	*****n***(%) in adherent group (***n*** = 8); ***p***-value**

Hypotension	3 (38); 0.24
≥3 organ systems in failure	5 (63); 0.03
Vascular access (>1 IV attempt)	2 (25); 0.76
Use of infusion pump	5 (63); 0.24
Appropriate initial fluid bolus (20 ml/kg over 15–20 min)	3 (38); 0.01

### Vascular Access

Delay in vascular access was seen in 7/35 (20%) patients in the non-adherent group. Two patients of our total of 43 (5%) patients received an intraosseous (IO) placement during their ER stay. To stratify the other 41 patients, an IV was established on the first attempt in 24/41 (59%) patients, on the second attempt in 4/41 (10%) patients, and on the third attempt in 4/41 (10%) patients. An IV access was already in place at triage in 9/43 (21%) patients.

### Fluid Administration

Delay in initiation of appropriate fluid bolus was seen in 2/35 (6%) patients in the non-adherent group. Administration of fluids using an infusion pump instead of manual push was reported in all of the patients in the non-adherent group and 5/8 (63%) of patients in the adherent group. Of the 43 (9%) patients, 4 received fluid via a rapid fluid push, and 2/43 (5%) patients did not receive a fluid bolus. A total of 10/43 (23%) patients were placed on IV infusions at 999 ml/h. Though 26/43 (60%) of patients received 20 ml/kg of fluid, the choice of infusing over 1 h (16/26, 62%) instead of 10–20 min was commonly observed. Appropriate resuscitative volume and rate of fluid bolus delivery were observed more frequently in the adherent group (3/8, 38%) versus the non-adherent group (2/35, 6%) (*p* = 0.01).

### AMDS Data

Accurate timing of vascular access, fluid and antibiotics were further elucidated using AMDS data. For the time from triage to fluids administered (median of 76 min with IQR 25–75% of 46.5–128 min), triage to IV placement was a median of 48.5 min (IQR 25–75% of 48–170 min), and IV placement to fluid administration was a median of 5 min (IQR 25–75% of 0–15 min). From triage to FLP was a median of 26 min (IQR 25–75% of 13–41.5 min), FLP to fluid order was a median of 5 min (IQR 75–25% of 0–63 min), and fluid order to administration was a median of 15 min (IQR 25–75% of 4–45.5 min) (Figure 2).

For the time from triage to antibiotics administered (median of 135 min with IQR 25–75% of 67.5–187.5 min), triage to antibiotic order was a median of 107 min (IQR 25–75% of 48–170 min) further broken down into triage to FLP which was a median of 26 min (IQR 25–75% of 13–41.5 min) and FLP to order entry which was a median of 93 min (IQR 25–75% of 18.75–141.5 min). Antibiotic order to administration time was a median of 19 min (IQR 25–75% of 6.3–33 min) and was also further broken down into antibiotic order to AMDS dispensation which was a median of 7 min (IQR 25–75% of 0–31.75 min) and AMDS dispensation to antibiotic administration which was a median of 17 min (IQR 25–75% of 13–30 min) (Figure 3).

Hypotension and three or more organ dysfunctions were more commonly observed in patients in the adherent group [3/8 (38%) and 5/8 (63%), respectively] as compared to patients in non-adherent group [10/35 (14%) and 8/35 (23%), respectively]. As compared to non-adherent group, a significantly larger number of patients in adherent group had >3 dysfunctional organ systems (*p* = 0.03). Of the 43 patients, there were four mortalities: 100% received inadequate fluid resuscitation, 2/4 (50%) received antibiotics within 1 h, 3/4 (75%) did not follow guidelines for vascular access either due to delay or multiple attempts before considering IO placement, and the fourth patient arrived with central access already in place.

## Discussion

Our data are concerning as they show that adherence to PALS guidelines for SS management is not being adequately followed at our institution (Figures 2 and 3) and that a number of factors are associated with non-adherence to guidelines, with many opportunities for improvement. Previous studies evaluating adherence to guidelines have shown similar data (3, 11). A myriad of studies in the adult literature have focused on factors and developed QI projects, interventions, nursing-teams, and other process improvements that have shown significant improvement in morbidity and mortality rates, and duration of hospital stay for adult patients (12–16). There is a paucity of data on factors leading to non-adherence to guidelines and measures to improve adherence in the pediatric population. This study is not the first to evaluate adherence to guidelines (3, 8, 17). It is, however, one of the first studies to focus on the *factors contributing to poor adherence* at a tertiary children’s hospital serving south Texas.

Adherence in our institution was first called into question after the realization that many patients were receiving fluid resuscitation with an infusion pump. We also observed that patients who were more ill-appearing tended to receive a more rapid evaluation and intervention in contrast to those patients who had smoldering sepsis. Given these observations we compiled charts of patients coded as SS and began a retrospective review. We were able to identify factors related to non-adherence using the EMR.

In our institution, as soon as the patient is first seen in the ER triage, a set of vitals are obtained and a triage score is assigned to indicate acuity of the patient. Then, the patient is moved to the main ER area where the ER attending sees the patient. The triage time was defined as the time spent in the ER triage before being seen by an ER attending. Time to vascular access was defined as the time when nurse recorded in the nursing documentation that an IV was placed. *Fluid- and antibiotic-order times* were defined as the respective time when orders were placed by the FLP and *fluid- and antibiotic-administration times* were defined as the respective times when fluid and antibiotic were started. In order to delineate factors associated with delays it was important to distinguish each individual time to determine areas of targeted improvement (Figures 2 and 3).

### Vascular Access

Adherence to the guideline recommending vascular access within 15 min with a one-time IV attempt before considering IO is low (20 and 25% of patients in the non-adherent and adherent groups, respectively). The time from triage to IV placement also appears to be the longest delay in the overall adherence to the guideline for fluid administration (Figure 2). There appears to be a hesitation in obtaining rapid vascular access via IO line placement when IV placement is difficult, especially in those patients who appear to be more stable. This reticence is not unique to our institution and has been noted in the Children’s Safety Initiative-Emergency Medical Services as one of the top three most challenging procedural skills in children among pediatric ER nurses (18). Only 2 of our 43 patients had an IO placed, with four patients undergoing a third IV-placement attempt. As none of the patients had undergone attempts to place an IV prior to having an IO, use of an IO appears to have been user-dependent, suggesting that the provider’s comfort in placing an IO guided its use. We suspect that this factor contributed to the repeated attempts at IV instead and likely contributed to the median of 48.5 min to vascular access in our patients (Figure 3).

### Appropriate Fluid Bolus Therapy

Frequently, fluid bolus was administered using an infusion pump (86%) instead of a manual push (9%), which is well described in the literature as only effective in those patients weighing less than 16 kg when attempting to do effective fluid resuscitation (8, 19). In the Stoner et al. prospective, randomized interventional trial, they determined that rapid fluid bolus is best achieved with either a pressure bag or push-pull method using the aggressive 2002 PALS guidelines of 20 ml/kg over 5 min, which can be adapted to the 2015 PALS less aggressive guidelines for fluid resuscitation (60 ml/kg over 60 min before inotropes) (6, 19). In our study, we did not explore the factors related to the use of infusion pump rather than manual push for fluid resuscitation, which was used in the majority of our patients, but potential reasons may be staffing issues or ER providers’ preferences. In a previous QI project to improve sepsis guideline adherence, this preference was also noted as an issue requiring a change in nursing culture toward manual push or rapid infuser only, which significantly improved timeliness of fluid resuscitation (triage to first bolus 72–22 min) (8).

### Antibiotic Timeliness

Pediatric Advanced Life Support guidelines recommend early administration of antibiotics in SS, and our institution currently struggles with this guideline due to multiple factors. Our study showed the longest delay tended to be between triage and administration of antibiotics (median of 135 min), with the longest subset delay being between triage to the antibiotic order being placed, indicating an issue with recognition of sepsis (Figure 3). With the advent of antibiotics readily available in the AMDS, once SS was recognized, obtaining antibiotics tended to be relatively quick (median of 7 min), however, the bulk of the antibiotic administration time (median of 17 min) appears to be related to preparation, which may be related to nursing antibiotic preparation skills.

### Severity of Illness

In our study, the adherent cohort was more likely to have hypotension (38 vs. 14%) and worsening systemic organ dysfunction (63 vs. 23%). This observation suggests that recognition of severe sepsis in relatively well-appearing patients may be delayed further. It may also largely explain why no difference in mortality rates was noted between patients in the adherent group versus the non-adherent group. Staff who cared for sicker patients tended to be more compliant with sepsis guidelines than those who cared for patients who appeared less ill. It is important to note, however, that of the four deaths that occurred during the study period, all of them received initial fluid bolus therapy over 1 h after triage and only two received antibiotics within the first hour of presentation. These factors further support the sepsis guidelines, especially the timely interventions for sepsis management.

Under-recognition of sepsis found in our study could be related to a variety of factors, some of which could be education and training on sepsis, retention of assessment skills, practical application of guidelines or lack of an objective scoring system or alert system. In the in-patient setting, early warning scores have been used as objective measures of the patient’s status to activate rapid response and codes, which provide opportunities for rapid assessment and rapid deployment of a series of interventions. A similar scoring system in triage may assist with early identification and intervention in sepsis, especially in those who are not hypotensive. Our emergency department uses the Emergency Severity Index scoring system for triage which bases triage on level of resources. It uses temperature for pediatric patients to help grade their severity but does not routinely use vital signs for all levels of triage severity and thus has the potential to miss SIRS criteria (20). Another potential solution is the protocol approach. When a protocol and order set were instituted in other QI projects, the expediency of antibiotics became standardized, resulting in more rapid administration and standardized selection (6).

As a result of this study, the creation of electronic alerts and a sepsis protocol have been integrated into our EMR. The electronic alert uses data recorded at the triage and if the patient’s triage assessment is concerning for sepsis, it triggers an electronic sepsis alert that enables ER providers initiate a prompt sepsis management in that patient. In the future, we hope to study the effect of a simulation-based sepsis education curriculum for all care-providers in the ER to help improve compliance with PALS sepsis-guidelines and outcomes in children with sepsis. Included in this curriculum will be training for nurses on push–pull fluid resuscitation and antibiotic preparation skills to improve efficiency and multitasking.

Multifaceted educational interventions have also been shown to shorten delays in antibiotic administration in children with SS when using workshops, meetings, posters, leaflets, email reminders, and online training simulation (21). In addition, recent studies at The Children’s Hospital of Philadelphia showed that algorithmic alerts integrated in the EMR based on vital-signs and high risk conditions in conjunction with serial physician judgment and “sepsis huddles” tend to produce the best sensitivity and specificity for SS (96.6–99.4% sensitivity and 83.3–99.1% specificity) encouraging the use of both staff training in conjunction with EMR alerts (22, 23).

This study highlights the importance of assessing the extent to which an institution is implementing guidelines and of addressing deficiencies to improve the management of our sickest patients. In the future, we hope to use a more protocolized approach to SS patients aimed to improve the morbidity and mortality associated with severe sepsis and SS in the pediatric population here in South Texas.

### Limitations

Our study is limited by its small sample size (*n* = 43) at a single center over the course of a year of study. The only way for our institution to determine patients presented with severe sepsis or SS is to use ICD-9 codes. As a result, another limitation of our study was the use of ICD-9 codes for “severe sepsis” and “SS” which were frequently a misnomer for patients as evidenced by the initial 58 patients who were categorized as such, ten of whom did not meet SIRS criteria during their stay in the ER. Our analysis did not include the patients who did not meet SIRS criteria. We also had to exclude patients who progressed to cardiorespiratory arrest during triage or ER evaluation since their management priority changed to cardiopulmonary resuscitation; and therefore, the only document available in these patients was their cardiac arrest/code sheet.

## Conclusion

Overall adherence to guidelines for management of sepsis was low. The factors associated with non-adherence to the guidelines were (a) more than one attempt at vascular access, (b) delay in antibiotic order entry, (c) fluid administration using infusion pump, and (d) presence of normal BP and fewer than three organ dysfunctions at time of presentation to the ER.

## Author Contributions

All the authors contributed equally.

## Conflict of Interest Statement

The authors declare that the research was conducted in the absence of any commercial or financial relationships that could be construed as a potential conflict of interest.
